# Adenosquamous Carcinoma of Vesicovaginal Fistula: A Rare Entity

**DOI:** 10.1155/2014/654638

**Published:** 2014-04-30

**Authors:** Rudresh Tabali, Aravind Ramkumar

**Affiliations:** Department of Surgery Oncology, Jawaharlal Institute of Postgraduate Medical Education and Research (JIPMER), Pondicherry 605006, India

## Abstract

A 56-year-old lady presented with a vesicovaginal fistula (VVF) along with past history of abdominal hysterectomy. Biopsy of the fistulous tract showed squamous cell carcinoma (SCC). Patient underwent radical cystourethrectomy, total vaginectomy, and bilateral pelvic lymph node dissection along with ileal conduit. The final histopathology report of the resected specimen showed adenosquamous carcinoma in VVF. As this is a rare entity, we are reporting this case.

## 1. Introduction

Adenosquamous carcinoma is a type of cancer that contains two types of cells: squamous cells and gland-like cells. It is frequently found in carcinoma of the colon, endometrium, and head and neck but less commonly in that of pancreas, skin, lung, cervix, and small intestine [1–4]. Adenosquamous carcinoma arising in VVF has not been reported in the literature.

## 2. Case Presentation

A 56-year-old lady came with history of dribbling of urine per vagina of three months duration. She also had history of previous abdominal hysterectomy six years back; details of surgery including indications, intraoperative findings, and histopathology of the specimen were unavailable. On examination, the patient had a fistulous opening of size 7 × 5 cm noted in the vault of vagina, with mild induration of surrounding region. CECT of abdomen and pelvis showed VVF with associated enhancing wall thickening of the bladder and vault around the fistula. Cystoscopy showed tumor with fistula in the posterior wall of bladder. Biopsy taken from the fistula showed evidence of squamous cell carcinoma.

**Figure 1:**
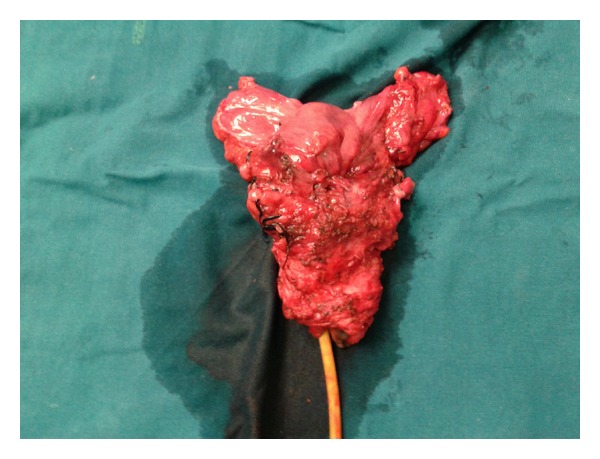
Specimen of radical cystourethrectomy + total vaginectomy.

After evaluation patient underwent open radical cystourethrectomy, total vaginectomy, and bilateral pelvic lymph node dissection along with ileal conduit in March 2013 (Figure 1). The postoperative course was uneventful.

**Figure 2:**
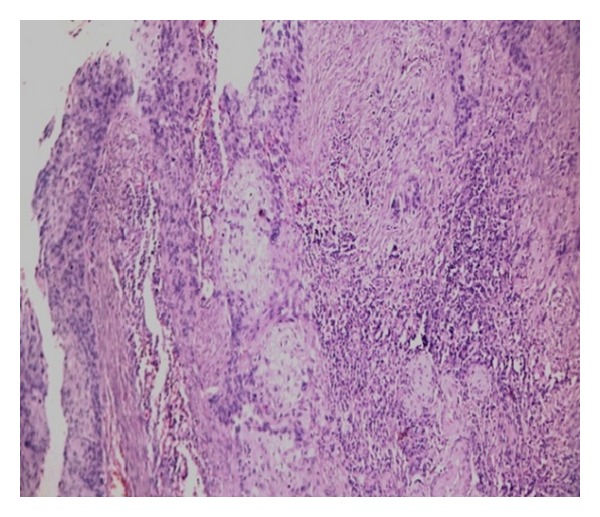
Islands of atypical squamous epithelium infiltrating underlying detrusor muscle with associated dense lymphocytic infiltrate (H&E stain, 100x).

**Figure 3:**
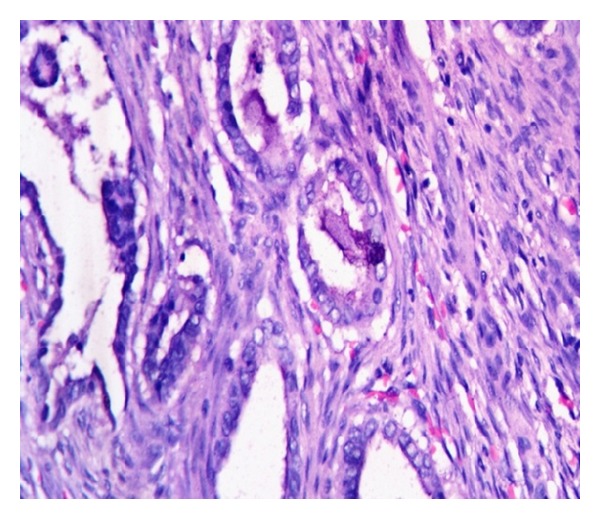
Neoplastic glandular elements with intraluminal mucin, infiltrating detrusor muscle (H&E stain, 400x).

Final histopathological examination of the resected specimen showed squamous cell carcinoma (SCC) of bladder, moderately differentiated, infiltrating muscularis propria up to adventitia (Figure 2). The bladder wall near the fistulous tract showed SCC in situ and subepithelial tissue showed adenocarcinoma, well differentiated with high ki 67 expression (Figure 3). The vaginal cuff showed SCC in situ changes. There was no evidence of human papillomavirus (HPV) infection like koilocytosis. All margins were free of tumor and a total of ten lymph nodes were harvested which were also free of tumor. The patient is on regular followup since surgery and has been free of recurrence till date.

## 3. Discussion

Vesicovaginal fistula is a fistulous communication between bladder and vagina. Etiology for VVF may be of benign or malignant origin. Prolonged obstructed labor remains the commonest cause of VVF in the developing world [5]. Other benign causes of VVF are gynecological surgeries like hysterectomy, pelvic irradiation, pelvic inflammatory diseases, uterine rupture, and so forth [6, 7]. Malignant causes of VVF are carcinoma of cervix, vagina, bladder, and endometrium [8]. A fistula that occurs in association with a malignancy of the female reproductive tract may be caused by a primary or recurrent tumor or may be a complication of surgery or radiation therapy [9].

Bladder tumors may also present with VVF if the tumor is located on the posterior wall. In the United States, primary bladder neoplasms account for 2–6% of all tumors. Urothelial carcinoma has a propensity for multidirectional differentiation, 90% of which are transitional cell carcinoma [10]. SCC accounts for 2–15% of bladder tumors with rates varying widely according to geographical location and adenocarcinoma represents less than 2%. Mesenchymal tumors represent the remaining 5% of bladder tumors, with the most common type being rhabdomyosarcoma and other types being paraganglioma, lymphoma, leiomyoma, and solitary fibrous tumor.

SCC of the bladder can be further classified as bilharzial and nonbilharzial based on the etiology of cancer. Bilharzial SCC occurs due to infection of* Schistosoma haematobium* which is endemic in Egypt and other African regions [11]. However, nonbilharzial SCC is associated with chronic irritation of bladder from urinary stasis due to bladder outlet obstruction, recurrent urinary tract infections, bladder stones, prolonged indwelling catheter, and cyclophosphamide exposure [12].

Adenocarcinoma of the bladder is characterized histologically by a pure glandular phenotype. These tumors are most often derived from the urothelium of the bladder (nonurachal adenocarcinoma) and less often arise from a remnant of the urachus (urachal adenocarcinoma) [13]. SCC and adenocarcinoma of bladder generally present at an advanced stage and carry poor prognosis [14, 15].

The primary site of malignancy in VVF in our case could be hypothesized to be from any of the following sites. Firstly, it could be a recurrent cervical squamous cell carcinoma, a recurrence after previous hysterectomy (of which reports are not available) as there is an evidence of SCC in situ in the vaginal cuff and there is also evidence of SCC in the posterior wall of the bladder. However to explain adenocarcinomatous change in the fistula would be difficult unless the primary in the cervix was of adenosquamous variety. The other possible site could be from the bladder as urothelium is known to differentiate into a wide variety of tissue types like SCC, adenocarcinoma, and so forth. Yet another possible site may be from adenosquamous carcinoma of the vagina, as few cases of this type have been reported by Sulak et al. [16]. However, no invasive component in the resected specimen of vagina on histopathology makes it an unlikely cause.

Cervical adenosquamous carcinoma originates from columnar cells of the cervical mucosa. It accounts for 3–5% of cervical carcinoma and it contains both adenocarcinoma and SCC components, formed through simultaneous differentiation of reserve cells towards adenocytes and squamous cells. A histopathological diagnosis of cervical adenosquamous carcinoma predicts poor outcome compared to that of pure adenocarcinoma type, especially in advanced stages [17].

## 4. Conclusion

Though vesicovaginal fistula is common, malignancy in vesicovaginal fistula is rare. We are reporting here a rare case of composite adenosquamous carcinoma in vesicovaginal fistula.

## References

[1] Haqqani MT, Fox H (1976). Adenosquamous carcinoma of the endometrium. *Journal of Clinical Pathology*.

[2] Masand RP, El-Mofty SK, Ma X-J, Luo Y, Flanagan JJ, Lewis JS (2011). Adenosquamous carcinoma of the head and neck: relationship to human papillomavirus and review of the literature. *Head and Neck Pathology*.

[3] Na YJ, Shim K-N, Cho MS (2011). Primary adenosquamous cell carcinoma of the pancreas: a case report with a review of the Korean literature. *Korean Journal of Internal Medicine*.

[4] Ngo N, Villamil C, Macauley W, Cole SR (1999). Adenosquamous carcinoma of the small intestine: report of a case and review of the literature. *Archives of Pathology and Laboratory Medicine*.

[5] Sjøveian S, Vangen S, Mukwege D, Onsrud M (2011). Surgical outcome of obstetric fistula: a retrospective analysis of 595 patients. *Acta Obstetricia et Gynecologica Scandinavica*.

[6] Duong TH, Gellasch TL, Adam RA (2009). Risk factors for the development of vesicovaginal fistula after incidental cystotomy at the time of a benign hysterectomy. *The American Journal of Obstetrics and Gynecology*.

[7] Stanojević D, Djordjević M, Martins F (2010). Repair of vesicovaginal fistula caused by radiation therapy with labia maiora skin flap. *Srpski Arhiv za Celokupno Lekarstvo*.

[8] Atuhairwe S, Busingye RB, Sekikubo M, Nakimuli A, Mutyaba T (2011). Urologic complications among women with advanced cervical cancer at a tertiary referral hospital in Uganda. *International Journal of Gynecology and Obstetrics*.

[9] Narayanan P, Nobbenhuis M, Reynolds KM, Sahdev A, Reznek RH, Rockall AG (2009). Fistulas in malignant gynecologic disease: etiology, imaging, and management. *Radiographics*.

[10] Johansson SL, Cohen SM (1997). Epidemiology and etiology of bladder cancer. *Seminars in Surgical Oncology*.

[11] El-Bolkainy MN, Mokhtar NM, Ghoneim MA, Hussein MH (1981). The impact of schistosomiasis on the pathology of bladder carcinoma. *Cancer*.

[12] Kassouf W, Spiess PE, Siefker-Radtke A (2007). Outcome and patterns of recurrence of nonbilharzial pure squamous cell carcinoma of the bladder: a contemporary review of the university of Texas M. D. Anderson cancer center experience. *Cancer*.

[13] Grignon DJ, Ro JY, Ayala AG, Johnson DE, Ordóñez NG (1991). Primary adenocarcinoma of the urinary bladder: a clinicopathologic analysis of 72 cases. *Cancer*.

[14] El-Sebaie M, Zaghloul MS, Howard G, Mokhtar A (2005). Squamous cell carcinoma of the bilharzial and non-bilharzial urinary bladder: a review of etiological features, natural history, and management. *International Journal of Clinical Oncology*.

[15] Gill HS, Dhillon HK, Woodhouse CRJ (1989). Adenocarcinoma of the urinary bladder. *British Journal of Urology*.

[16] Sulak P, Barnhill D, Heller P (1988). Nonsquamous cancer of the vagina. *Gynecologic Oncology*.

[17] Farley JH, Hickey KW, Carlson JW, Rose GS, Kost ER, Harrison TA (2003). Adenosquamous histology predicts a poor outcome for patients with advanced-stage, but not early-stage, cervical carcinoma. *Cancer*.

